# Supplementary material for: Signal coverage approach to the detection probability of hypothetical extraterrestrial emitters in the Milky Way
Source: Sci Rep. 2017 Apr 12;7:46273. doi: 10.1038/srep46273 (PMC5388861; doi:10.1038/srep46273)
Supplement: Supplementary Information [file srep46273-s1.pdf]

Supplementary Information

Signal coverage approach to the detection probability of hypothetical extraterrestrial emitters in the Milky Way

Claudio Grimaldi

Laboratory of Physics of Complex Matter, Ecole Polytechnique Fédérale de Lausanne, Station 3, CH-1015, Lausanne, Switzerland

e-mail: claudio.grimaldi@epfl.ch

Age scale of detectable signals for unbounded distributions of the shell thickness

Equations 4 and 5 of the main text give the detection probability for a spherical signal with shell thickness distributed up to a maximum value Δ_M. As long as we consider bounded distribution functions of the shell thickness [$\rho_\Delta(\Delta)$], the value of Δ_M gives a definite age of the oldest detectable signal, $t_M = (R_M + \Delta_M)/c$, which allows us to interpret Eq. 4 as the conditional probability that the Earth intersects a signal given that it has been emitted within a time t_M before present.

In the case of unbounded distributions of the shell thickness, we can still define an age scale of the oldest detectable signal if $\rho_\Delta(\Delta)$ is such that $\bar{\Delta} = \int_0^\infty d\Delta \Delta \rho_\Delta(\Delta)$ exists. In this case, if we assume that the distribution of the outer radii [$\rho_R(R)$] can be approximated by a constant, Eq. 4 still gives the detection probability. In order to identify the age scale of the oldest emitted signal, we consider the probability distribution of the time of emission of the detectable signals (that is, those signals that have outer radii smaller than $R_M + \Delta$)

$$\rho_{\text{emission}}(t) = \int_0^\infty d\Delta \rho_\Delta(\Delta) \int_0^{R_M+\Delta} dR \rho_R(R) \delta(t - R/c) \cong \rho_R [1 - \theta(ct - R_M) P_\Delta(ct - R_M)], \tag{S1}$$

where we have set $\rho_R(R) \cong \rho_R$, $\theta(x)$ is the Heaviside step function, and $P_\Delta(x) = \int_0^x d\Delta \rho_\Delta(\Delta)$ is the probability that the shell thickness is smaller than x. The age scale of the oldest detectable signals can be identified as the time t_M at which $\rho_{\text{emission}}(t_M)/\rho_{\text{emission}}(0)$ is sufficiently close to zero. For example, by asking that $\rho_{\text{emission}}(t_M)/\rho_{\text{emission}}(0) = 0.01$ we plot in Fig. S1 $\Delta_M = ct_M - R_M$ as a function of the standard deviation (sd) for a lognormal distribution of Δ

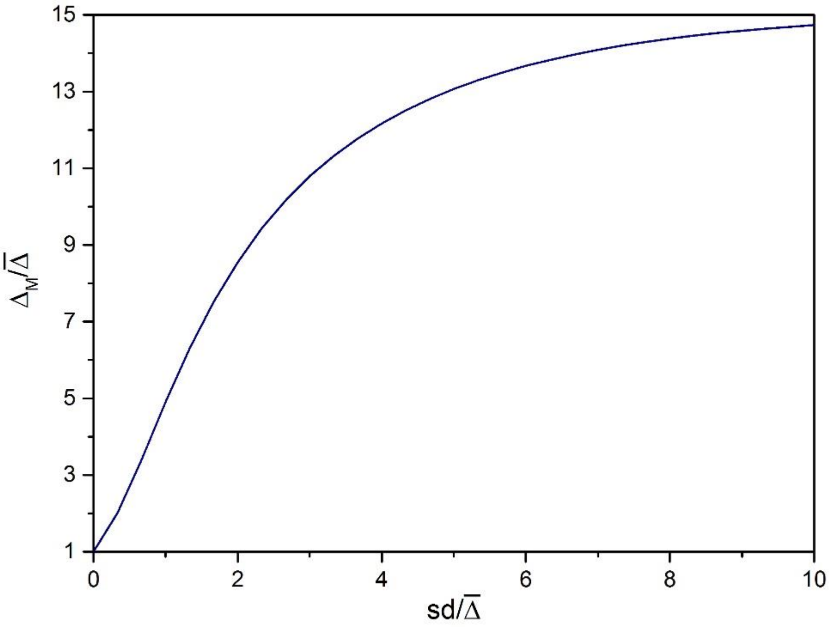

Figure S1. Largest shell thickness (Δ_M) as a function of the standard deviation (sd) for a lognormal distribution of the thickness of a spherical shell signal. Δ_M is obtained by requiring that $\rho_{\text{emission}}(t_M)/\rho_{\text{emission}}(0) = 0.01$ where $t_M = (\Delta_M + R_M)/c$ is the corresponding age of the oldest emitted signal.

with mean shell thickness $\overline{\Delta}$. From Fig. S1 we see that $\Delta_M \approx 15$ kly for $\overline{\Delta} = 1$ kly and $\text{sd} = 10$ kly, which corresponds to an age scale of the oldest signal of about 100'000 years.

Models for the emitter distribution function

In the main text, we have considered a model of the emitter distribution function with cylindrical symmetry [$\vec{r} = (r, z, \phi)$],

$$\rho_E(\vec{r}) = \rho_0 r^m e^{-r/r_s - |z|/z_s}, \tag{S2}$$

where the parameters m, r_s, and z_s have been chosen so to simulate two models for the galactic habitable zone (GHZ) in the Milky Way: one in which the GHZ follows the distribution of stars in the galactic thin disk, and the other in which the GHZ is an annular region in the thin disk located at about 22 kly from the galactic center. Here, we show some additional results obtained by varying the radius of the annulus so to analyze the effect on the probability $\pi(R_o)$ of finding an emitter within a radius R_o from the Earth, which is defined in Eq. 3 of the main text. Note that the detection probability of a single emitter is proportional to $\pi(R_o)$ (Eq. 5 of the main text).

Along the radial distance, Eq. S2 has its maximum at $r = mr_s$. We calculate $\pi(R_o)$ by keeping r_s fixed at 1 kpc ($= 3.26$ kly) and by varying m from $m = 0$ up to $m = 15$. In this way, the

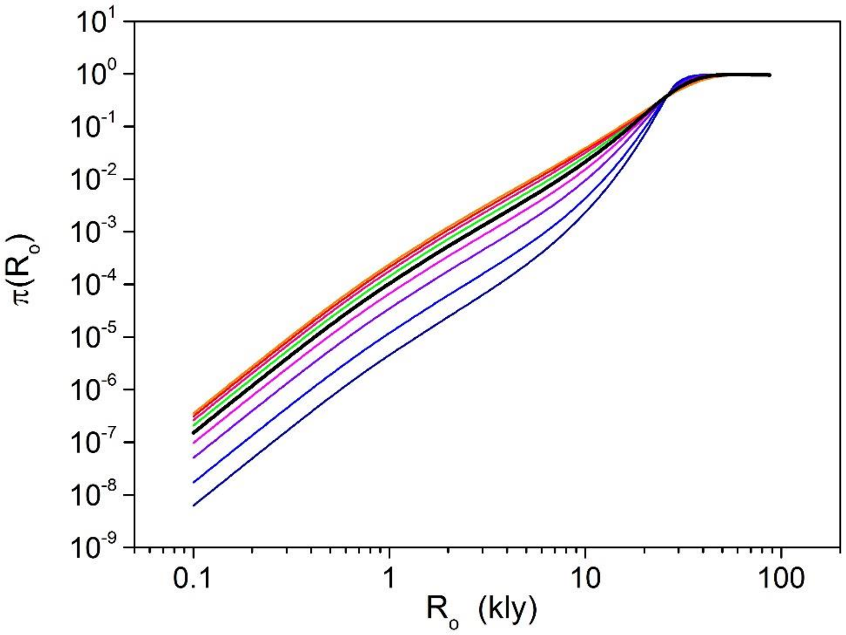

Figure S2. Probability $\pi(R_o)$ of finding an emitter with a radius R_o from the Earth obtained from Eq. S2 for $z_s = 0.52\,\text{kly}$, $r_s = 3.26\,\text{kly}$, and $m = 0, 1, 3, 5, 7, ..., 15$ from bottom to top. The thick black line denotes the case $m = 7$ considered in the main text.

maximum of $\rho_E(\vec{r})$ moves from the galactic center to the periphery of the galaxy. In calculating $\pi(R_o)$, we place the Earth on the galactic plane at a radial distance of 27 kly from the galactic center. Figure S2 shows the results for $m = 0, 1, 3, 5, 7, ..., 15$ (from bottom to top) as a function of the observable radius. The thick black line denotes the case for $m = 7$ which corresponds to the annular GHZ discussed in the main text. From the figure we see that a shift of the maximum of the emitter distribution from the center to the periphery of the galaxy may result in a change of about one order of magnitude for fixed R_o. As already pointed out for the cases discussed in the main text, $\pi(R_o)$ is proportional to $R_o{}^3$ for $R_o < 2\,\text{kly}$ and the value of m has only a weak effect for $R_o > 20\,\text{kly}$.
